# Mutations in *Helicobacter pylori* infected patients with chronic gastritis, intestinal type of gastric cancer and familial gastric cancer

**DOI:** 10.1186/s13053-024-00282-8

**Published:** 2024-06-12

**Authors:** Andrzej Hnatyszyn, Marlena Szalata, Aleksandra Zielińska, Karolina Wielgus, Mikołaj Danielewski, Piotr Tomasz Hnatyszyn, Andrzej Pławski, Jarosław Walkowiak, Ryszard Słomski

**Affiliations:** 1 Independent Public Health Care Centre in Nowa Sol, Multispecialty Hospital, Chalubinskiego 7, Nowa Sol, 67-100 Poland; 2 Department of Biochemistry and Biotechnology, Poznań University of Life Sciences, Dojazd 11, Poznań, 60-632 Poland; 3 Department of Biotechnology, Institute of Natural Fibres and Medicinal Plants National Research Institute, Wojska Polskiego 71B, Poznań, 60-630 Poland; 4 Department of Pediatric Gastroenterology and Metabolic Diseases, Poznan University of Medical Sciences, Szpitalna 27/33, Poznań, 60-572 Poland; 5 Faculty of Medicine, Pomeranian Medical University, Rybacka 1, Szczecin, 70-204 Poland; 6 Institute of Human Genetics, Polish Academy of Sciences, Strzeszyńska 32, Poznań, 60-479 Poland; 7 Department of General, Endocrinological Surgery and Gastrointestinal Oncology, Institute of Surgery, Poznan University of Medical Sciences, Przybyszewskiego 49, Poznań, 60-355 Poland

**Keywords:** *Helicobacter pylori*, Chronic gastritis, Intestinal type of gastric cancer, Familial gastric cancer, DNA variants, Molecular diagnostics, *TP53*, *NOD2*

## Abstract

**Background:**

Development of sequential changes of mucous leading to gastric cancer and familial cases of gastric cancer of intestinal type is widely connected with *Helicobacter pylori* infections. In this study we analysed variants of genes involved in cancerogenesis and inflammatory processes of intestines in patients infected with *H.pylori*. Our goal was to test whether mutations in these genes predestinate to development of gastric cancer, and whether there is a genetic factor that makes it more likely for infections with *H.pylori* to cause gastric cancer. As infections with *H. pylori* are relatively common, discovering such genetic predispositions could be used for establishing risk-groups and for planning treatments.

**Methods:**

Our studies cover analysis of variants in genes involved in cancerogenesis: *TP53* (rs11540652, rs587782329, COSM10771), *MSH2* (rs193922376), *MLH1* (rs63750217), and inflammatory processes of intestine: *NOD2* (rs2066847, rs2066842), *IL1A* (rs1800587) and *IL1B* (rs1143634) from *H.pylori-*infected patients.

**Results:**

Mutations were more common in the group of patients with gastric cancer of intestinal type and familial cases of gastric cancer in comparison with patients with chronic gastritis, chronic atrophic gastritis, intestinal metaplasia, dysplasia or gastric cancer (p-value = 0.00824), with the prevalence of p53 mutations in patients with familial gastric cancer vs. patients with other changes of mucosa (p-value = 0.000049). Additionally, gastric cancer patients have mainly genotype TT or CT of the rs2066842 variant of the *NOD2* gene.

**Conclusions:**

The lack of statistically significant changes of other interleukin genes involved in inflammatory processes may suggest the presence of *H.pylori* infection as a potential trigger for the development of the inflammatory process of the mucosa, leading through microbiota dysbiosis to the development of enteric gastric cancer. Mutations in analysed genes correlated with more severe mucosal changes, with a much more frequent presence of *TP53* gene mutations, with a limited presence of other mutations in the familial history of gastric cancer.

## Background

Gastric cancer (GC) is the fourth most common type of cancer contributing to cancer mortality worldwide and is the fifth most common cause of new cases of cancer according to GLOBOCAN 2020 [1]. Gastric cancer consists of heterogeneous group of different types of gastric cancer, depending also on genetic background with different types of histological and molecular classifications [2]. The most common histological type of gastric cancer, gastric adenocarcinoma, consists of two pathological variants: intestinal and diffuse. Early stages of stomach cancer may be asymptomatic. Gastric cancer may be influenced by hygiene, healthy eating, and Epstein-Barr virus or *Helicobacter pylori* bacterium infections [3–7]. Treatment depends on the stage of the disease, but surgical tumour resection with regional lymphadenectomy remains the gold standard [8]. Intestinal and diffuse types of gastric cancer have different genetic backgrounds and need to be treated in different ways [9]. Stomach cancer is most common in East Asia, Japan, Korea and East China, with China accounting for more than 40% of the world’s cases [10]. It is also widely present in Latin and South America and Eastern Europe but is less common in Southern Asia, North America, North and East Africa, Australia and New Zealand [7, 8, 11]. The occurrence of gastric cancer depends on the interaction of environmental and genetic factors. *H.pylori* infection is considered a major etiological factor in the development of gastric cancer and peptic ulcer disease [10]. The individual lifetime risk of developing gastric cancer is assumed to be 1.5–2% in *H.pylori*-infected individuals, which is significant with more than 1 million cases per year and a high mortality rate. Most stomach cancers are related to *H.pylori* infection. There is a need to develop guidelines for gastric cancer with a family history, while guidelines for hereditary familial gastric cancer are available. Due to the synergistic effect of *H.pylori* infection and family history on the increased risk of gastric cancer, *H.pylori* eradication can be recommended in all infected individuals with a family history of gastric cancer. It is assumed that the eradication of bacteria is worth starting at the age of 20, before the appearance of precancerous changes including significant gastric atrophy and intestinal metaplasia [12] and is mainly transmitted in childhood by mothers [10]. Eradication of *H.pylori* significantly decreases incidences of gastric cancer [5, 13].

It is estimated that this bacterium, which is a confirmed oncogenic factor, lives in the gastrointestinal tract of more than half of human population in the world. This percentage varies depending on the level of economic development of a given country - in developed countries, it may be as low as 24%, while in developing countries it may even reach 70% [10]. The effectiveness of *H.pylori* eradication is up to 90%, and the problem of bacterial resistance to antibiotics is a major clinical problem. In Eastern Europe, Poland is one of the countries with a high infection rate - in Lower Silesia, 56.7% of strains of this bacteria are resistant to metronidazole, and 55.2% to clarithromycin [14]. According to research, the percentage of infected people in our country reaches approx. 84% among adults and approx. 32% among people under 18 years of age [14].

Risk factors also include a diet low in fresh fruits and vegetables, a high-salt diet, poorly preserved food, excessive alcohol consumption, and obesity. Other risk factors may include smoking, occupational risk factors such as dust, nitric oxide, N-nitrous compounds and radiation, or an Epstein-Barr virus (EBV) infection. It is also assumed that the risk of developing gastric cancer increases 2–3 times in patients with a family history of gastric cancer [8, 15].

Lauren’s histological classification of gastric cancer is the most commonly used, which distinguishes two main subtypes: the intestinal form and the diffuse form [2, 16]. The intestinal form of gastric cancer is associated with chronic atrophic gastritis and intestinal metaplasia, while the diffuse form is associated with normal gastric mucosa. Routine diagnostics include upper gastrointestinal endoscopy and radiological examinations. In the case of diffuse gastric cancer, screening for mutations in the *CDH1* gene is performed, while in the case of the intestinal type, there is no specific molecular background [11].

The onset of the intestinal type of gastric cancer is considered to be associated with the development of sequential stages ranging from non-atrophic gastritis, atrophy, intestinal metaplasia and dysplasia (intraepithelial neoplasia) to gastric cancer. In contrast, the etiology of diffuse gastric cancer remains unknown. The presence of gastric cancer in first-degree relatives is one of the most important risk factors for the development of gastric cancer. Patients are often at increased risk of developing gastric atrophy or intestinal metaplasia earlier than developing gastric cancer [17].

The main goal of studies included an analysis of molecular-genetic disturbances of frequencies of mutations of genes involved in cancerogenesis like *TP53* (rs11540652, rs587782329, COSM10771), *MSH2* (rs193922376) and *MLH1* (rs63750217) mostly associated with hereditary nonpolyposis cancer and *NOD2* gene (rs2066847) involved in lesions of intestine and inflammatory bowel diseases. Due to inflammatory processes, three polymorphisms were also analysed including *NOD2* (rs2066842), *IL1A* (rs1800587) and *IL1B* (rs1143634) genes variants.

All patients showed infection with *H.pylori*. For analysis, tissues and peripheral blood cells of patients with chronic gastritis, chronic atrophic gastritis, intestinal metaplasia, dysplasia, gastric cancer and familial cases of gastric cancer from Western Poland were selected. *H.pylori* infection was confirmed by histological analysis and urease test. Screening procedures involved single-strand conformational polymorphism and heteroduplex analysis. Samples indicating the presence of aberrant DNA fragments were subjected to direct sequencing and pyrosequencing. All analyses and selection of patients and control groups were described earlier [18–20].

## Materials and methods

Studies were performed using a well-defined patient group selected from 1430 patients with different stages of intestine mucosa and gastric changes and also cases of gastric cancer intestinal type (17 patients) and familial gastric cancer (12 patients). For analysis, 40 patients with chronic gastritis, 36 patients with chronic gastritis with atrophy, 17 patients with chronic gastritis with intestinal metaplasia and 21 patients with chronic gastritis with dysplasia (Tables 1, 2 and 3) were selected. The control group used in the study included two subgroups: (1) 13 patients with dyspepsia without lesions and without infection of *H.pylori* in the gastric mucosa (control group) (Tables 2 and 3) and (2) 100 people from the population group (Table 4), which were selected as described earlier [18, 19]. The TIBCO Software Inc. (2017) Statistica (data analysis software system), version 13. http://statistica.io was used for statistical analysis. Non-parametric methods were used to verify the hypotheses: the chi-square test (in the case of small expected numbers, the Yates correction was used), the Mann-Whitney U test and the Spearman’s rank correlation coefficient. The significance level was assumed to be α = 0.05. The results were considered statistically significant when the calculated test probability *p* (computer significance level) met the inequality *p*-*value* < 0.05. All patients were confirmed to be infected with *H.pylori*. Methods of detection of *H.pylori* presence, gastroscopic and molecular analyses were presented in other papers prepared by our group [18–20]. The following approvals were obtained from the Bioethics Committee of the Poznan University of Medical Sciences: 871/09, 442/13 and 995/17.

**Table 1:** Characteristics of patients and population groups

Group of patients	Number of patients	Gender		Age (years)
		Females	Males	
Control group, without lesions and infection of *H.pylori* in gastric mucosa	13	7 (54%)	6 (46%)	16–57 37.8 (± 12.9)
Chronic gastritis	40	20 (50%)	20 (50%)	16–75 35.6 (± 14.6)
Chronic gastritis with atrophy	36	19 (53%)	17 (47%)	11–76 41.0 (± 18.5)
Chronic gastritis with intestinal metaplasia	17	8 (47%)	9 (53%)	13–81 38.4 (± 17.2)
Chronic gastritis with dysplasia	21	12 (57%)	9 (43%)	17–87 52.5 (± 18.7)
Gastric cancer intestinal type	17	5 (29%)	12 (71%)	42–84 62.2 (± 12.5)
Familial gastric cancer intestinal type	12	7 (58%)	5 (42%)	18–63 41.5 (± 18.0)
Population group	100	50 (50%)	50 (50%)	21–30 25.5 (± 2.9)

**Table 2:** Molecular characteristics of patients. Mutations

Group of patients	Number of patients	*MSH2* rs193922376	*MLH1* rs63750217	*NOD2* rs2066847	*TP53* rs11540652 rs587782329 COSM10771
Control group, without lesions and infection of *H.pylori* in gastric mucosa	13	-	-	-	-
Chronic gastritis	40	1 (3%)	2 (5%)	7 (18%)	6 (15%)
Chronic gastritis with atrophy	36	1 (3%)	1 (3%)	2 (6%)	4 (11%)
Chronic gastritis with intestinal metaplasia	17	1 (6%)	1 (6%)	2 (12%)	5 (29%)
Chronic gastritis with dysplasia	21	3 (14%)	-	2 (10%)	6 (29%)
Gastric cancer intestinal type	17	1 (6%)	4 (24%)	3 (18%)	1 (6%)
Familial gastric cancer intestinal type	12	-	-	1 (8%)	8 (67%)

**Table 3:** Molecular characteristics of patients. Polymorphisms

Group of patients	Number of patients	Alleles	*NOD2* rs2066842	*IL1A* rs1800587	*IL1B* rs1143634
Control group, without lesions and infection of *H.pylori* in gastric mucosa	13	CC	10 (77%)	6 (46%)	7 (54%)
		CT	1 (8%)	4 (31%)	5 (38%)
		TT	2 (15%)	3 (23%)	1 (8%)
Chronic gastritis	40	CC	19 (48%)	21 (53%)	18 (45%)
		CT	15 (38%)	12 (30%)	16 (40%)
		TT	6 (15%)	7 (18%)	6 (15%)
Chronic gastritis with atrophy	36	CC	24 (67%)	18 (50%)	22 (61%)
		CT	7 (19%)	13 (36%)	9 (25%)
		TT	5 (14%)	5 (14%)	5 (14%)
Chronic gastritis with intestinal metaplasia	17	CC	9 (53%)	4 (24%)	7 (41%)
		CT	6 (35%)	9 (53%)	5 (29%)
		TT	2 (12%)	4 (24%)	5 (29%)
Chronic gastritis with dysplasia	21	CC	10 (48%)	8 (38%)	12 (57%)
		CT	6 (29%)	9 (43%)	6 (29%)
		TT	5 (24%)	4 (19%)	3 (14%)
Gastric cancer intestinal type	17	CC	5 (29%)	9 (53%)	9 (53%)
		CT	8 (47%)	6 (35%)	6 (35%)
		TT	4 (24%)	2 (12%)	2 (12%)
Familial gastric cancer intestinal type	12	CC	1 (8%)	1 (8%)	3 (25%)
		CT	2 (17%)	11 (92%)	9 (75%)
		TT	9 (75%)	-	-

**Table 4:** Molecular characteristics of the population group

Population	Number of patients	Alleles	*NOD2* rs2066842	*IL1A* rs1800587	*IL1B* rs1143634
Polish population	100	CC	68	48	63
		CT	28	41	28
		TT	4	11	9

## Results

Mutations were more common in the group of patients with higher severity of symptoms – gastric cancer intestinal type and familial cases of gastric cancer in comparison with patients with chronic gastritis, chronic atrophic gastritis, intestinal metaplasia, dysplasia and gastric cancer (chi-square Pearson’s 6.980973, df = 1, *p*-value = 0.00824). Prevalence of p53 mutations in patients with familial gastric cancer vs. patients with other changes of mucosa was observed (67% vs. 17%, respectively, chi-square 16.4936, *p*-value = 0.000049). The familial gastric cancer patients group encompasses also only one mutation of the *NOD2* gene, and other mutations like *MSH2* and *MLH1* were not observed.

We have observed different distribution of genotypes of rs2066842 *NOD2* gene variant for minor mucosa lesions in comparison with gastric cancer and familial gastric cancer cases (CC 54.39%, TT 15.79%, CT 29.82% vs. CC 20.69%, TT 44.83% and CT 34.48% respectively, chi-square Pearson’s 14.67573, df = 2, *p*-value = 0.00065). It is consistent with the observation of the change in the genotype distribution of this *NOD2* variant from the predominance of the CC genotype in controls to an increase in the incidence of TT in patients (CC 69.03%, TT 5.31%, CT 25.66% vs. CC 47.55%, TT 21.68% and CT 30.77%, respectively, chi-square Pearson’s 17.38210, df = 2, *p*-value = 0.00017). No correlation between controls and patients or gastric cancer cases for variant rs1800587 of *IL1A* gene was observed (chi-square Pearson’s 1.806898, df = 2, *p*-value = 0.40517 for all patients, chi-square Pearson’s 2.274738, df = 2, *p*-value = 0.32066 for minor changes and chi-square Pearson’s 3.320390, df = 2, *p*-value = 0.19010 for gastric cancers, respectively). Observed changes in allele frequency in a variant of the *IL1A* gene are not statistically significant. Similar situation may be shown for rs1143634 of *IL1B* gene for all patients (chi-square Pearson’s, 4.311039, df = 2, *p*-value = 0.11584), patients with minor changes (chi-square Pearson’s, 3.857192, df = 2, *p*-value = 0.14535), and cancer patients vs. healthy controls, respectively (chi-square Pearson’s, 5.257258, df = 2, *p*-value = 0.07218).

Genotype CT of the *NOD2* gene rs2066842 variant was more frequent in patients with diagnosed mutations of *TP53*, *MSH2*, *MLH1* or *NOD2* genes, respectively (chi-square Pearson’s 11.42658, df = 2, *p*-value = 0.00330). We have shown a statistically significant increase in symptoms with the patient’s age, Spearman’s correlation of average strength was observed (*R* = 0.39; *p*-value = 0.000397). The Mann-Whitney U test confirmed that the group with more severe symptoms was older (Z=-3.4; *p*-value = 0.000501). Patients with gastric cancer were significantly older than patients with minor changes in the mucosa (54 vs. 41 years, *p*-value = 0.000732). The statistically significant earlier incidence of gastric cancer in familial cases is also significant compared with sporadic cases of gastric cancer (62 vs. 42 years, *p*-value = 0.001042), which is consistent with other analyses showing the influence of family cases on earlier onset of gastric cancer.

## Discussion

Gastric cancer is considered as a worldwide disease and is usually associated with acquired chronic gastritis, in most cases due to *Helicobacter pylori* infection. A small percentage of these relate to familial cases, and some of them can be linked to specific germline mutations [21]. The influence of genetic background on gastric cancer was suggested already in 1914 by Theodor Boveri [22]. The epigenetic basis of cancer is now accepted. Gastric cancer is a highly heterogeneous disease with a variety of molecular and genetic characteristics, including several chromosomal changes, epigenetic changes and the influence of miRNA molecules [23]. The lack of conclusive data on gastrointestinal cancer is confirmed by extensive studies including the determination of chromosome instability and copy number variability, the effect of single variants or the prediction of some risk factors for gastric tumours and the impact of *H.pylori* infection [23]. That’s why in our research, we focused on the influence of the genetic background while assuming the presence of *H.pylori*. Genomic variants pose a risk to different types of cancer. Single nucleotide polymorphisms (SNPs) are common genetic variations and differ from mutations due to occurrence with a frequency of 1% or more in the population [23].

Of different types of gastric cancer only hereditary diffuse gastric cancer (HDGC) is known to be inherited [24] and is associated with the presence in approximately 45% of familial cases with a mutation in the E-cadherin gene (*CDH1*), but the genetic background of other cases remains unknown. Hereditary diffuse gastric cancer and familial cases of gastric cancer are reported in 10% of all gastric cancer cases, therefore in most gastric cancer cases, we are not able to identify molecular factors involved in cancerogenesis [23, 24]. Our studies also showed that gastric cancer of intestinal type and familial cases lack the most common variant which may be strictly associated with a strong influence on the development of cancer. Still, the main factor remains infection with *H.pylori*, which in Poland affected over 80% of adults [14] and in analysed patients was confirmed in all cases (100%) by urease test [18, 19].

*H.pylori* infection is etiologically associated with gastritis with progression to gastric cancer, it is assumed that the elimination of *H.pylori* significantly reduces the incidence of gastric cancer [6, 25–28]. Retrospective studies of over 371,000 veterans with confirmed *H.pylori* infection showed a significantly higher risk of gastric cancer in racial and ethnic minorities and smokers. Only successful eradication of *H.pylori* reduced the risk of gastric cancer [29]. *H.pylori* infection is an important factor in the development of gastric cancer as a result of the cascade of gastric precancerous conditions. Chronic infection is also associated with genetic and epigenetic changes. It is assumed that the eradication of *H.pylori* infection significantly reduces the risk of developing gastric cancer [30]. *H.pylori* infection is important due to the frequent occurrence of familial infections, which favours the development of familial cases of gastric cancer, while the presence of *H.pylori* is often the only determinant of an increased risk of cancer [31]. Detection and treatment of *H.pylori* infection are worthwhile in patients with a family history of GC in the United States [32]. Studies indicate that clearing *H.pylori* infection in first-degree relatives who have developed gastric cancer has reduced the risk of developing stomach cancer [33]. Currently, it is indicated that the dysbiosis of the gastric microbiome is important factor in the development of gastric cancer, especially since the presence of *H.pylori* is detected in asymptomatic cases [34]. Dynamic changes in the microbiome were observed depending on the developmental stage, ranging from gastritis through intestinal metaplasia to GC, and in addition to *H.pylori*, the number of lactic acid bacteria, *Fusobacterium* and *Leptotrichia* increases [35, 36]. Such an approach may contribute to the emergence of new strategies for the prevention and treatment of GC [34].

New cases of gastric cancer associated with *H.pylori* are most common in East Asia responsible for the increased incidence of gastric cancer. *H.pylori* colonizes the gastric mucosa thanks to the interaction of virulence factors (adhesion, translocation, inflammation and infectivity of the host gastric epithelium: cytotoxin-associated gene *CagA*, vacuolation cytotoxin A *VacA*, high-temperature requirement A factor *HtrA*, blood group antigen-binding adhesin *BabA*, sialic acid-binding adhesin *SabA*, gamma-glutamyl transpeptidase *GGT*, outer inflammatory protein A *OipA*), host factors (pro-inflammatory cytokines including tumour necrosis factor-α TNF-α, interleukin-1 β IL-1β, interleukin-8 IL-8, interleukin-10 IL-10, nuclear factor kappa B NFkB, cyclooxygenase-2 COX-2, tumour suppressor gene *TP53*, reactive oxygen species ROS) and environmental factors (dietary salt and cigarette smoking) [37].

In our studies, however, we focused only on the analysis of genes that may facilitate the development of *H.pylori* infection and changes in the gastric epithelium leading to the development of gastric cancer. Nevertheless, the great importance of *H.pylori* infection in these processes is indicated, especially since *H.pylori* infections persist in familial systems.

Although the mechanism by which *H.pylori* influences the development of gastric disease is not fully understood, it is assumed that there is a relationship between *H.pylori* virulence factors and the host inflammatory/immune response. Pro-inflammatory and anti-inflammatory cytokines are involved in the induction of other cytokine expression, cell proliferation, differentiation, necrosis and apoptosis and are modified by the presence of *H.pylori* [38]. Interleukin 6 (IL-6) and interleukin 8 (IL-8) are believed to appear early in the cascade of inflammatory molecules, and *H.pylori* also stimulate the production of interleukins 1β (IL-1β), 2 (IL-2), 10 (IL-10), 12 (IL-12), interferon (IFN) and tumour necrosis factor (TNF) in the gastric immune response [25, 39, 40]. Ultimately, gastric endothelial cell inflammation caused by *H.pylori* can lead to gastric cancer [23].

Selected variants of the interleukin 1β promoter are believed to influence the inflammatory response of the gastric mucosa to *H.pylori* infection, may increase the risk of gastric cancer [41–43], and their effects may be modified by genetic and environmental factors. These sequence variants do not appear to be responsible for the occurrence of familial high-penetration gastric cancer [21].

Interleukin 1β (IL-1β) plays a key role in the inflammatory pathway - when induced by *H.pylori* infection, it inhibits gastric acid secretion, which promotes *H.pylori* colonization and leads to more severe gastritis [44, 45]. However, there is no clear indication of the involvement of a specific variants of this interleukin with gastric cancer. *H.pylori* infection stimulates an immune response involving interleukin-1β (IL-1β), leading to strong expression of IL-1β, inhibition of gastric acid secretion, methylation and GC-related gene dysfunction, and angiogenesis. The process of IL-1β maturation in macrophages, neutrophils and dendritic cells is mediated by the Nod-like domain of the pyrin receptor containing 3 (NLRP3) inflammasomes. While the mechanism of IL-1β release through the cell membrane is not fully understood, a strong association with *H.pylori* infection makes this pro-inflammatory cytokine an important component in the initiation of inflammation and progression of gastric cancer, providing new targets for early detection and better treatment of gastric cancer [45].

The *IL1B* gene has three clinically important DNA variants: the base transition of CT in IL-1B-511 (rs16944), the base transition of TC in IL-1B-31 (rs1143627) and IL-1B-3954 (rs1143634), all of them strongly associated with increased production of pro-inflammatory cytokines, hypochlorhydria, and an increased risk of GC, mainly intestinal, in Caucasians but not Asians or Hispanics [46–49]. The T allele of IL-1B-511 increases the risk of gastric cancer, especially in the combination of the T allele and virulence markers (cagA positive, vacAs1 and vacAm1) [50]. Children with homozygous polymorphic alleles 511 (rs16944) and − 31 (rs1143627) may be exposed to relatively severe histological changes in the gastric mucosa [51]. Other studies indicate that the polymorphic alleles T rs16944 and C rs1143627 increase the risk of gastric cancer in the Chinese population, which is further enhanced by presence of *H.pylori* [52]. Zabaglia et al. in 2016, showed the relationship between other genotypes of interleukin-1β variants − 511T/T (rs16944) and − 31 C/C (rs1143627) with gastritis and the presence of *H.pylori* in Brazilian children (9.41 ± 4.29 years). The authors suggested that interactions between these polymorphisms affecting changes in the activity of the pro-inflammatory cytokine IL1β and *H.pylori* may be predictors of the risk of gastritis and early development of gastric disease, but they did not substantiate more advanced inflammation of the mucosa [43]. On the other hand, studies in a specific Indian population showed that IL-1B-511*T carriers homozygous for the IL-1RN short allele (IL-1RN*2/*2) were more likely to develop gastritis after infection with Asian cagA strain of *H.pylori* [53].

A meta-analysis by Zhang and coworkers [54] indicated that the c.315 C > T IL1B variant (rs1143634) was borderline significant with an increased risk of cancer. The aggregate results were influenced by individual studies, so more studies should be performed to assess the role of the c.315 C > T IL1B polymorphism in cancer etiology. Also, our previous studies showed that the presence of CT and TT genotypes of the IL1B rs1143634 gene variant was statistically significant [18]. However, after including the group with familial gastric cancer, no statistical significance was observed in the frequency of individual alleles, although the values were approaching statistical significance (gastric cancer and familial cases of gastric cancer vs. control *p*-value = 0.07218).

Pleiotropic effects of IL-1β variants in cancer may be confirmed because, in addition to the main pro-tumour activity, it can also have anti-tumour effects. It is assumed that the level of IL-1β produced, the type of producing cells, the microenvironment (immune cells or fibroblasts), the cancer stage and the anti-cancer therapies used may be involved in the divergent effects of IL-1β. More research is required to clarify these points, including the influence of *H.pylori* [23, 55]. In any case, the use of IL-1β blockers should be carefully considered in the clinical context to ensure the best treatment for patients [56].

The meta-analysis of *IL1B* gene variants (IL1B-511 C/T, IL1B-31 C/T, IL1B + 3954 C/T) performed for *H.pylori*-infected patients showed that after analysis using 9606 cases and 5654 controls only IL1B-511 C/T polymorphism was correlated with the risk of *H.pylori* infection [57]. A study conducted in the Chinese population of variants of interleukin 1 A (*IL1A*) and meta-analysis of *IL1A* 4-bp insertion/deletion polymorphism (rs3783553) showed a reduction by the rs3811047 G > A variant in the probability of development of gastric cardiac adenocarcinoma [58] and lack of activation of gastrointestinal cancer by rs3811047 G > A variant [59]. In this study, as in ours, no correlation between polymorphism IL1A (*IL1A*) rs1800587 C > T and gastric symptoms was found. But the authors claim that a higher number of analysed groups may verify the presented association [58].

A family history of gastric cancer increases the occurrence risk of stomach mucosa changes from a normal to precancerous status covering atrophic gastritis through intestinal metaplasia, and dysplasia until gastric cancer and decreases the age of onset [17]. A similar situation was observed also for our groups. Among the environmental factors of gastric cancer, *H.pylori* infection cannot be ignored - it is considered to be one of the etiological factors responsible for at least 75% of gastric cancer cases worldwide [8, 13]. This is also consistent with our data, as all analysed patients had confirmed bacterial infections. *H.pylori* infection may be indicated as the main factor influencing the development of sporadic gastrointestinal cancers which did not show a significant genetic basis for carcinogenesis. Also, infection with *H.pylori* and familial intestinal gastric cancer may be connected with the earlier age of onset of cancer, which is consistent with the available data [8, 60]. *H.pylori* is believed to lead to chronic gastritis, which, through a process called the Correa’s cascade, can lead to atrophic gastritis, intestinal metaplasia, and dysplasia into gastric cancer and potentially influence the development of cancer in other tissues [61]. As not all *H.pylori* carriers develop gastric cancer, the involvement of the host genetic background has been suggested [55]. Genome-wide association studies (GWAS) revealed several single nucleotide polymorphisms (SNPs) associated with *H.pylori* colonization and eradication and the development of intestinal metaplasia. Among the genes involved in these processes are the autophagy and inflammasome mediators *ATG16L1*, *IL1B* and *CARD8* and also pattern recognition receptors (PRRs) and Toll-like receptors (TLRs) [55].

Familial gastric cancers account for approximately 10% of cases, with most familial cases in regions with low gastric cancer incidence likely due to hereditary pathogenic mutations, but the genetic basis of familial intestinal gastric cancer remains genetically unexplained [60]. Compared to sporadic gastric cancers, next-generation sequencing has shown that they may be polygenic. In studies conducted by Carvahallo et al. (2021), 12 rare variants were found in patients with familial intestinal gastric cancer only in 11 probands. Patients also developed gastric cancer at least 10 years earlier than in sporadic cases and shared more *TP53* gene germline variants in cells [62], and microsatellite instability was observed in 38% of birth-cell tumours [63]. Also in our studies, the presence of *TP53* gene variants was observed only in familial intestinal gastric cancer, while changes in the *MLH1* and *MSH2* genes were observed only in sporadic cases of intestinal cancer. The observed differences may suggest the influence of germline and somatic mutations on the occurrence of gastric cancer.

The presence or absence of TP53 active or inactive types may be used as biomarkers of new gastric cancer subtypes [23, 64] and may be independent of other subtypes: mesenchymal type, which included diffuse type of gastric cancer or microsatellite unstable (MSI) type, which included hypermutated intestinal-type gastric cancer [3, 23, 64]. Genetic changes in the tumour suppressor gene *TP53* are assumed to be significant in early and advanced cancer. The most common is loss of gene function through loss of heterozygosity, mutations and changes in the level of methylation. The most common mutations of the *TP53* gene concern codons 175, 245, 248, 249, 273 and 282, and are associated with the risk of gastric cancer; similar changes were also observed in our cases. The risk of cancer also increases in the case of a reading frame disorder, the occurrence of nonsense and silent mutations. Since risk factors may also include the occurrence of single nucleotide polymorphisms in combination with interactions between genes and the environment (ethnicity, geographical location, *H.pylori* infection, smoking), age, bile or acid reflux, we cannot rule out a significant influence of the *TP53* gene based on the relevant studies for the development of intestinal-type gastric cancer [65].

*TP53* mutations are found in 50% of intestinal-type gastric cancer, mainly characterized by chromosomal instability (CIN, 49%), aneuploidy and focal amplification, and they were observed to affect receptor tyrosine kinases, VEGFA and cell cycle mediators. *TP53* mutations are frequently observed in the non-cancerous gastric mucosa with *H.pylori* infection, and various chromosomal aberrations are also present in gastric adenoma as part of the cascade leading through atrophy, metaplasia, dysplasia to gastric cancer [3]. *TP53* mutations are more associated with *H.pylori* infection, the main cause of the development of intestinal subtype GC alongside dietary behaviour, which may be connected with ethnic differences in the risk of development of gastric cancer [7].

*H.pylori* infection leads to the activation of the *NOD1* and *NOD2* genes; several variants of which have been observed to be associated with gastric cancer in different populations [55]. Particular attention is paid to *NOD2* due to its involvement in inflammatory bowel disease (IBD). It is assumed that reduced activity associated with NOD1 and NOD2 promotes bacterial survival, and an excessive inflammatory response may result in carcinogenesis associated with the condition inflammatory. In our previous studies, we confirmed the link between the rs2066842 (c.802 C > T) *NOD2* variant and the risk of gastric cancer for the Polish population [20]. Also, changes in rs2066847 (1007 fs) and rs2066845 (G908R) in the *NOD2* gene lead to a reduction in bacterial removal efficiency in IBD patients [55]. It is assumed that *NOD2* mutations limit the ability of the NOD2 protein to bind bacterial polysaccharides, which is associated with an increase in NF-κB activity and an overactive pro-inflammatory response. This phenomenon may occur in chronic gastritis caused by *H.pylori*, leading to an increased risk of gastric cancer, but the available data are inconclusive. This is due to the study of different groups of patients with various inflammatory lesions of the gastric mucosa, small numbers, analysis of different variants of the *NOD2* gene and incomplete information on *H.pylori* infection [66]. The main limitation of most studies on the association of *NOD2* gene mutations with gastric cancer was the lack of data concerning *H.pylori* infection status. Such a situation is presented in the article by de Almeida [66], in which the R702W rs2066844, G908R rs2066845 and L1007fsinsC rs2066847 variants of the *NOD2* gene were analysed, mainly in patients with non-ulcer dyspepsia without cases of gastric dysplasia and gastric cancer, with the authors pointing to the potential involvement of other genetic factors that can modify the immune response.

Inflammation is assumed to be one of the factors associated with tumour development [67], which may be associated with *H.pylori* infections. The occurrence of cancer is also influenced by the genetic background of the host, pathogen, diet and environmental factors, including population conditions, hence it is difficult to identify the direct causes of cancer. More studies are needed to elucidate the importance of DNA variants, the relationship with *H.pylori* infection, and the development of intestinal metaplasia and GC [55]. Of course, the development of advanced gastric cancer is influenced by numerous, not fully understood molecular mechanisms. In addition to the selected genes analysed in this paper, it is worth paying attention to the participation of long non-coding RNAs (lncRNAs), which can regulate target genes at the level of transcription, post-transcription and translation by modifying mRNA, miRNA and proteins. Therefore, attention should be paid to the need to use the widest possible research that can shed light on the development of gastritis and gastric cancer after *H.pylori* infection, to fully define the microenvironment associated with this process, and to identify new biomarkers and therapeutic targets [68].

The tendency to develop gastric cancer is certainly influenced by polymorphisms of genes involved in inflammatory processes, including a pleiotropic proinflammatory cytokine IL-1β and IL-1RN, TNF-α, IL-8, IL-10, proinflammatory cyclooxygenase enzymes (COX), modulation of acids secretions, generation of oxidative stress, immune response to *H.pylori*, modification of the barrier function of apical-junctional complexes, as well as regulation of the apoptosis process. The influence of environmental factors should not be forgotten, including excessive salt intake, parasitic infections (e.g. *Helminth*), dietary supplementation with antioxidants, and smoking [69]. Critical host responses that affect the progression of *H.pylori*-induced carcinogenesis include gastritis and decreased acid secretion. The risk of developing gastric cancer depends on many factors, including the influence of *H.pylori*, the patient’s genotype and environmental exposure [3, 69]. Despite such a diversified share of individual factors, it seems fully justified to indicate the significant impact of *H.pylori* infection on the development of familial cases of gastric cancer.

## Conclusions

Gastric cancer of the intestinal type is connected with different genetic and environmental factors, mainly inflammatory changes of mucus. The main risk factor is *H.pylori* infection, present in all analysed cases. The presence of mutations in selected genes correlated with more severe mucosal changes, with a much more frequent presence of *TP53* gene mutations with a limited presence of other mutations in the familial history of gastric cancer. The greater share of *TP53* gene variants and earlier development of cancer in familial cases is consistent with the observations of other research groups. Moreover, genotype TT or CT of rs2066842 *NOD2* gene polymorphism was much more common in gastric cancers than in other groups of patients. No statistically significant contribution of polymorphisms of interleukin 1-α and 1-β genes or *NOD2* gene mutation (rs2066847) was observed. This suggests a shortening of the development period of the individual leading to a faster onset of cancer. Due to the lack of molecular studies of *H.pylori* in our studies, the influence of the bacterial molecular background on the development potential of the tumour cannot be indicated.

## Data Availability

The datasets used and/or analyzed during the current study are available from the corresponding author on reasonable request and subject to institutional agreements for data sharing.
